# Tuberculosis and the Dairy-Farm

**Published:** 1899-04-15

**Authors:** Harold Sessions

**Affiliations:** Lecturer Oxford University Extension, Lecturer Sussex County Agricultural College


### TUBERCULOSIS AND THE DAIRY-FARM.

By Harold Sessions, F.R.C.V.S., F.H.A.S., Lecturer Oxford University Extension, Lecturer Sussex County Agricultural College.

At the present moment, owing to the action of certain exalted and learned gentlemen, based on the obscure labours of many unknown workers, the tuberculous cow has obtained a recognition and publicity long wanted and long denied. With the publicity there has come a feeling that a very large proportion of our home-grown milk is contaminated with tubercle bacilli, and that it is unsafe to drink. Particular stress has been and is laid on the large death-rate of children at the period when they are chiefly subsisting on cow's milk, and parents have been advised, in order to avoid the danger of tuberculosis, to feed the children on boiled or sterilised milk. I should like to point out that in avoiding one danger there is a fear of running into another. The boiling and sterilising of milk alters and does away with some of the antiscorbutic properties it possesses, and children are not likely to develop the same healthy and strong tissues on such milk that they would on a good pure unboiled milk. The questions of the infectiousness of tuberculous milk and the identity of bovine and human tuberculosis do not come within the scope of this paper, and they must be taken as admitted facts. The question is rather why has cow's milk, which is an article of universal diet, come under the suspicion of contamination, and is it possible that such suspicion can be removed?

### Tuberculosis a House Disease.

With bovines we find the agencies which control the spread of tuberculosis are the same as those existing with humans. It has been proved that the principal factors in spreading the disease are contagion and housing. Among wild animals in a state of nature tuberculosis is almost unknown. Among wild animals kept in confinement, as in the case in zoological gardens, tuberculosis commits great ravages. Among our domestic animals, sheep (whose lives are spent entirely out of doors) are rarely affected with tuberculosis, and the authentic instances of disease occurring in them are exceedingly few, yet there are many millions of sheep in the country. Among horses, who spend a large portion of their lives out of doors, we get isolated cases of tuberculosis, but they are few and far between. Dogs are to a limited extent affected with tuberculosis, and cats are fairly frequently found to be victims of this malady. Pigs, we find, are sometimes affected in considerable numbers, and where a pig is diseased it is usually found that a number of others living on the same premises are affected, but the primary affection is usually due to humans or bovines. Amongst cattle there are certain breeds in which it is rare to find cases of tuberculosis, and when it is found it is because the cattle are not being kept according to the "customs of the country"; such, for example, as when the black cattle of Wales are brought from their native hills to the somewhat limited accommodation of the London cow-stall.

In other breeds of cattle we expect to find a certain percentage of the animals affected, and this percentage varies from about 5 per cent. to (in extreme cases) 70 or 80 per cent. The conclusion one is forced to accept, after carefully considering the many details involved in such an inquiry, is that as far as bovine tuberculosis is concerned, although the disease is almost exclusively confined to certain breeds, yet it is not the breed of cattle that is an important factor, but the manner in which their lives are spent.

### The Warmth of Cow Houses.

All the breeds of cattle in which tuberculosis exists to an appreciable extent are breeds which are kept on account of their being able to supply large quantities of milk, and the object of their owners is to get the largest quantity of milk in the shortest possible time from the cows. This means that these cows must be fed on highly stimulating and milk producing foods, and that they must be kept in houses where the temperature is above 50 deg. F. In winter this is accomplished by either using artificial heating apparatus, or by keeping a number of cows close together without much air space or ventilation. It is needless to say the latter is the method which is largely adopted. In some cases we find that the conditions of farming are such that it is impossible from a commercial point of view to turn the cows into the pastures during any portion of the year, and they are tied up in sheds all the year round, never going out, except, perhaps, for a daily drink of water at the farm pond, and for a few weeks into a straw yard, just before they are due to calve. It is amongst cattle such as these that tuberculosis chiefly exists. The conditions of overcrowding, want of ventilation, air space, exercise, are potent accessory causes, and the presence of the bacillus tuberculosis soon causes many of the animals to be affected. But though a number of the animals are affected, the great majority are affected only in some of the internal glands or organs of the body, and the udder is free from disease. Experiment has proved that unless the udder is affected the milk from a tuberculous cow is harmless. Fortunately, only some 3 per cent. of the cows that are diseased are affected in the udder; and, still more fortunately, after a time the gland that is affected ceases to give milk and secretes a watery fluid. Even after we have made this allowance the fact remains that there are a certain percentage of cows with diseased udders whose milk is being used by human beings, and which undoubtedly causes disease and death. It is these cows which are casting the suspicion on what is otherwise a most nutritious food, and we may well ask ourselves, Where are these cows to be chiefly found, and what means can we adopt to stop their supplying milk to the community?

### The Commercial Side of Milk Production.

Dairy farming is carried on as a commercial undertaking and not as a philanthropic agency, and we cannot place any restrictions on the industry except such as are actually warranted by considerations of public health. Anything that is required more than this will have to be in the nature of a business arrangement between the public and the dairy farmers. It is often contended that the keeping of cattle in stalls is unnatural, that the cows should always be kept in the pastures, and that we should then get healthier cattle and a more wholesome milk supply. This is undoubtedly the case, and the nearer we keep the modern cow (which in itself is an artificial product) in a state approaching that which the wild cow lived in, the better it will be for the health of the cow and human beings. Unfortunately it is impossible to do this as completely as we desire.

### The Demands of the Public.

The public demands a certain regular quantity of milk for its breakfast and for its tea. It does not matter to the public if a sudden frost comes on, and every cow in the county which yesterday gave three gallons of milk, to-day only gives two gallons owing to the climatic change. It would be quite useless for the dairy farmer to explain that, as he wished to keep his cows healthy, he left them out of doors, and that for the next month or so every individual's baby which had been having a pint of milk daily must now manage with three-quarters of a pint, as his cows require the food which might have gone to produce milk to keep themselves warm. The quantity of milk must be had, and the only way to get it is to place the cows where they will feel the sudden changes of temperature, to which our island is subject, as little as possible. This, briefly, is the reason why cows are kept in sheds. It being admitted that it is necessary to keep cows in houses, and that such cows are the ones which are chiefly the subjects of tuberculosis, the further question arises, Can the conditions under which such cows live be regulated so as to ensure a pure milk supply?

### The Necessity for Fresh Air.

There is no doubt in my own mind that such conditions can be ensured with benefit to the public and the owners of the cows. These conditions will first of all consist in allowing all cows to spend the greater portion of the summer, at least, out of doors. It will be necessary to gradually alter many cows' sheds, introducing more windows, more air space, and better ventilation, so that the housed cattle, instead of breathing and rebreathing each other's breath, shall be supplied with fresh uncontaminated air. This, in many cases, means a large capital outlay on the part of property owners which, at the present price of milk, they do not incline to face. They say, and there is a good deal of strength in their argument, that until recently medical science taught that tuberculosis was an hereditary disease and not a contagious one. Now, they say, medical science teaches tuberculosis is a contagious disease, and what has taken scientific men years to grasp, we are asked to act upon suddenly and without preparation and at enormous expense.

### The Farmers' Protest.

For these reasons any very sudden or drastic interference with the dairy trade would be resented and lead to a great deal of difficulty. Already farmers all over England have united and joined associations to oppose any legislative interference with what they consider their just rights. Farmers are, however, alive to the value of an extra penny a gallon for their milk, and if the public are willing to pay a fair price for a good article they can undoubtedly have it. If the public say they wish their milk from cows which are kept in well ventilated sheds, if they desire to have milk from cows that are free from tuberculosis they can have it, but at present they will have to pay a trifle more for such milk; it costs more to produce and it is worth more money.

### The Tuberculin Test.

It has been established that cows which are injected under certain definite conditions with tuberculin will, if tuberculous, have a decided rise in their temperature during the succeeding twenty-four hours, while cows which are healthy are not in any way affected. By this means of diagnosis a herd of cows may be freed from this terrible scourge, and some public institutions and private people are now arranging to have their milk only from cows which have been so tested and are proved healthy. A great impetus would be given to the freeing of our herds from tuberculosis if medical men would use their influence to see that their milk and the milk of institutions came from herds from which the tuberculous cows had been eliminated, and a guarantee was given that all the fresh cows that entered the herd were previously tested with tuberculin and certified free from the disease. The cows should be under competent veterinary inspection at regular intervals, an inspection which should, if properly conducted, be serviceable to the dairy farmer as well as the public.

### Foul Sheds.

In many parts of England a great improvement in the ventilating and cleanliness of cowsheds has been introduced during the last few years, and this advance in veterinary hygiene has been particularly advocated by many members of my profession. At the same time there are cow sheds kept in a most filthy and insanitary condition, sheds into which it is impossible to go without feeling discomfort through the foul state of the air and the want of ventilation. It has been my experience that among dairy farmers the men whose sheds are so foul and filthy are the men with the least education, men who in the course of a few years we shall find it impossible to replace. On the other hand, we have dairy farmers of education who employ many thousands of pounds capital in their businesses, and who in general information and scientific knowledge are far ahead of the average business or commercial man.

### Public Apathy.

These dairy farmers have been complaining somewhat bitterly that the medical profession have alarmed the public about their milk supplies and yet will not help them when they place a pure article on the market. One dairy company I know have been offering to supply milk for some years from cows kept under the best sanitary conditions, which cows had been subjected to the tuberculin test, and the public and medical men in the vicinity were notified of the fact, but there was no demand for such milk. Another dairy company made arrangements to get a supply of good Jersey milk from cows also kept under model conditions, which herd is guaranteed to be free from any tuberculous cow. Before signing the contract the dairy company sent a circular to each of its customers—who are numerous—asking if any of them preferred to have this milk to that produced in the ordinary way, with the result that there was not a single response. Had the medical profession and public shown that they were really interested in the question of a pure milk supply in these cases and in other similar ones there is no doubt an impetus would have been given to rival dairy companies to do likewise. As one might expect, as this subject has become such an acute and public question, various means are devised of obtaining certificates as to the purity of the milk.

### Specious Certificates.

Perhaps the most specious and plausible of these is the certificate of a microscopic examination of the milk. This is now being foisted on the public by one or more of the larger dairy companies, and there is a medical association which sends round circulars offering to examine your milk microscopically and report as to the presence of tubercle bacilli. As I have already pointed out, the number of cows giving tuberculous milk is not more than one in every two hundred milking cows, and as the tubercle bacilli are difficult to detect in the milk of one cow, except in advanced cases, it is obvious that when the milk of such a cow is mixed with two hundred more cows' milk, that even after separating the milk you may examine hundreds of slides without finding any positive evidence of tuberculosis, and yet the milk would be dangerous. Such microscopical examination, besides being ineffective and misleading, is likely to produce a feeling of security where there is no security, and may prevent a thorough and beneficial inspection of the cows. At present, and I say advisedly at present, because I think there are signs of the conditions of the dairy trade altering, the only safe means is to test a herd of cows with tuberculin, and separate the healthy from the diseased, at the same time periodically inspecting the cows, paying particular attention to the state of the udders and mammary lymphatic glands. By this means we may free a herd from diseased animals, but it must not be forgotten that to keep them free great attention must be paid to give adequate air space in the cow sheds, and to see that there is proper ventilation.

There is no reason why even in winter a cowshed should not smell as sweet as an ordinary living room, and elsewhere[1] I have published figures showing that for commercial purposes cows are kept in sheds which are perfectly sweet and pure, and yet which give the required amount of shelter and warmth. In such sheds unless the disease is extraneously introduced, there is little danger of its making any headway. In 1895 I read a paper before an association of dairy farmers, pointing out that it was only a matter of time before this question became a public one, and I urged them to take time by the forelock, and be prepared to face and deal with the question. There are two paragraphs in that paper which I should like to reproduce.

### Fresh Air for Cows.

The first is with regard to the beneficial effects of fresh air for cows, even for those which are diseased, and reads: "I believe that large numbers of cattle recover after an attack of tuberculosis, and these animals will remain with the portion of their body attacked by the disease injured and altered to the end of their lives. But the actual disease has been overcome. Let us take as an example of this a strong young cow, and place her in a warm unventilated cow shed, feed her on forcing food, and tie her up in contact with a cow suffering from tuberculous pneumonia. The chances are that, with a constitution lowered by unhealthy surroundings taking in a large number of tubercle bacilli, she will be attacked, to a certain extent, by the disease, and if the conditions are continued she will become in time badly affected. If before the disease has advanced far she is taken out of this cow shed, placed in the open air, and fed on good nourishing (but not stimulating) food, particularly food of an oily nature, the chances are the disease will be stopped, and will not develop further unless she is again subjected to unhealthy surroundings."

### Consumptive Cowmen.

The second paragraph is one that will probably be corroborated by many country practitioners: "I have been particularly struck during the last few years with the large percentage of cowmen who are apparently affected in various degrees with phthisis, and I am convinced that their continual presence and expectoration amongst cattle and pigs is prejudicial to the animals. Men who are affected in this way should, for their own sake and that of the community, be employed on work which is unconnected with dairy stock." Hospitals and all large public institutions have a very great power for good in this matter. If they will insist that all their milk is from cows which have been properly inspected, and which have been subjected to the tuberculin test and are free from the disease; if they will insist on the cow sheds being up to the requirements of the Royal Commission on Tuberculosis with regard to light, air space, and ventilation; if they are prepared to recognise that milk is a variable fluid, and that a good quality milk may be cheaper than an inferior quality; if they are willing to give a higher price for such milk, making it worth the while of the dairy farmer to produce it, then a step will be accomplished towards freeing our herds from a disease which is as disastrous to bovines as it is to humans.

[1] "Cattle Tuberculosis." By Legge and Sessions. (Baillière, Tindall, and Cox.)

